# Trivalent Ions and Their Impacts on Effective Conductivity at 300 K and Radio-Protective Behaviors of Bismo-Borate Glasses: A Comparative Investigation for Al, Y, Nd, Sm, Eu

**DOI:** 10.3390/ma14195894

**Published:** 2021-10-08

**Authors:** Ghada ALMisned, Huseyin O. Tekin, Ghaida Bilal, Antoaneta Ene, Gokhan Kilic, Shams A. M. Issa, Merfat Algethami, Hesham M. H. Zakaly

**Affiliations:** 1 Department of Physics, College of Science, Princess Nourah Bint Abdulrahman University, Riyadh 11671, Saudi Arabia; gaalmisned@pnu.edu.sa; 2 Medical Diagnostic Imaging Department, College of Health Sciences, University of Sharjah, Sharjah 27272, United Arab Emirates; u17104268@sharjah.ac.ae; 3 Medical Radiation Research Center (USMERA), Uskudar University, 34672 Istanbul, Turkey; 4 Center for Advanced Materials Research, Research Institute of Sciences and Engineering, University of Sharjah, Sharjah 27272, United Arab Emirates; 5 INPOLDE Research Center, Department of Chemistry, Physics and Environment, Faculty of Sciences and Environment, Dunarea de Jos University of Galati, 47 Domneasca Street, 800008 Galati, Romania; 6 Department of Physics, Faculty of Science and Letters, Eskisehir Osmangazi University, 26040 Eskisehir, Turkey; gkilic@ogu.edu.tr; 7 Physics Department, Faculty of Science, University of Tabuk, Tabuk 47512, Saudi Arabia; sh_issa@ut.edu.sa; 8 Physics Department, Faculty of Science, Al-Azhar University, Assiut 71524, Egypt; 9 Physics Department, Faculty of Science, Taif University, Taif, P. O. Box 11099, Taif 21944, Saudi Arabia; M.algethami@tu.edu.sa; 10 Institute of Physics and Technology, Ural Federal University, 620002 Ekaterinburg, Russia

**Keywords:** bismo-borate glasses, trivalent ions, rare-earth, effective conductivity, Py-MLBUF, radiation shielding

## Abstract

We aimed to determine the contribution of various trivalent ions like Al and rare-earths (Y, Nd, Sm, Eu) on resistance behaviors of different types of bismo-borate glasses. Accordingly, eight different bismuth borate glasses from the system: 40Bi_2_O_3_–59B_2_O_3_–1Tv_2_O_3_ (where Tv = Al, Y, Nd, Sm, and Eu) and three glasses of (40Bi_2_O_3_–60B_2_O_3_; 37.5Bi_2_O_3_–62.5B_2_O_3_; and 38Bi_2_O_3_–60B_2_O_3_–2Al_2_O_3_) compositions were extensively investigated in terms of their nuclear attenuation shielding properties, along with effective conductivity and buildup factors. The Py-MLBUF online platform was also utilized for determination of some essential parameters. Next, attenuation coefficients, along with half and tenth value layers, have been determined in the 0.015 MeV–15 MeV photon energy range. Moreover, effective atomic numbers and effective atomic weight, along with exposure and energy absorption buildup factors, were determined in the same energy range. The result showed that the type of trivalent ion has a direct effect on behaviors of bismo-borate glasses against ionizing gamma-rays. As incident photon energy increases, the effective thermal conductivity decreases rapidly, especially in the low energy range, where photoelectric effects dominate the photon–matter interaction. Sample 8 had the minimum heat conductivity at low photon energies; our findings showed that Eu-reinforced bismo-borate glass composition, namely 40Bi_2_O_3_–59B_2_O_3_–1Eu_2_O_3_, with a glass density of 6.328 g/cm^3^ had superior gamma-ray attenuation properties. These outcomes would be useful for the scientific community to observe the most suitable additive rareearth type and related glass composition for providing the aforementioned shielding properties, in terms of needs and utilization requirements.

## 1. Introduction

Over the past few decades, radiation has become one of the most significant research topics. Over time, scientists have gained a better understanding of the potential for biological harm caused by various forms and characteristics of radiation. The most widely used classification system for categorizing radiation according to its potential to cause damage to living tissue divides it into two groups: ionizing and non-ionizing radiation. Ionizing radiation has come a long way in terms of widespread use and widely spreading its destructive effects on biological structures and living cells through direct or indirect effects [1]. It is important to protect people from the harmful effects of ionizing radiation. The word “radiation safety” has gained even more significance as a result of the application of radiation sciences in a variety of high-tech fields. For several years, certain traditional nuclear shielding materials have been used in some radiation fields based on use attributes [2]. Because of its low cost, easy availability, and ability to be shaped into any shape or form, concrete is the most commonly used shielding material [3,4] in nuclear reactors and cyclotrons. Concrete, however, has a number of drawbacks, which include cracks that appear after prolonged use, and the presence of water in concrete that reduces the material’s density and structural strength. Moreover, concrete is opaque by nature, making it difficult to see through. In the recent years, researchers have been trying to investigate the features of materials that can be utilized as a replacement for concrete and lead [5]—ones that are eco-friendly, highly dense, and chemically homogenous. Various materials, such as compounds, steel [6,7], and alloys [8,9,10,11] have been chosen and tested for their shielding applications in order to investigate the desired features. The findings revealed that strong, nontransparent concrete and lead outperform, prompting scientists to research glass systems to serve as radiation shielding materials. The precise composition of a glass can differ to meet the needs of certain applications. A typical glass can be described as clear, non-toxic, and usable with advanced material characteristics for a variety of purposes. Recent attention has been focused on glass samples doped with rare-earth elements, which show promise for a variety of applications, including optical amplifiers, laser instruments, and radiation shields. Glass samples with a higher concentration of bridging oxygen are critical for radiation shielding and mechanical properties [12,13,14]. The high density and optical transparency of glasses containing heavy metals such as Eu (Z-63), Sm (Z-62) and Nd (Z-60) make them ideal for radiation shielding applications [15,16,17]. Aluminum oxide and yttrium serve as modifiers due to their elemental mass fractions in glass samples. Bismuth oxide, on the other hand, has a significant density and refractive index advantage [18,19,20]. Yttria, a boro-bismuthate [21,22]-doped material that is used to control the arrangement of glass, is more valuable than borate or phosphate glass [23,24,25]. Additionally, it exhibits exceptional chemical and photochemical stability, in addition to highly evolved properties. The literature review, on the other hand, showed that the importance of Al_2_O_3_ has been investigated in terms of its critical properties. In a study, Popov et al. have compared thermal annealing of the F-type center in Al_2_O_3_ single crystals irradiated with heavy ions and neutrons [26]. Moreover, Kotomin et al. have investigated the kinetics of F center as well as colloid creation in Al_2_O_3_ [27]. As is well recognized, Al_2_O_3_ is of considerable importance due to its material characteristics. In another study, Tucker et al. have investigated the effects of neutron-irradiation on Al_2_O_3_ [28]. The present concept of these promising investigations has prompted us to do further research on the impact of other reinforcement types, including Al_2_O_3_, on the gamma-ray shielding properties of bismo-borate glasses. The aim of this study is to look into the nuclear shielding properties of various trivalent element oxides like Al_2_O_3_ and some rare-earth oxide-doped (Y^3+^, Sm^3+^, Nd^3+^, Eu^3+^) glass samples, using the Python multilayered buildup factor (Py-MLBUF) online platform [29] for energies between 0.015 and 15 MeV. Boro-bismuthate was chosen as the basic glass sample for this study due to its exceptional shielding and clarity properties. It should be noted that the results of this study will be applicable to the field of glass literature, particularly radiation shielding. In addition, the obtained findings can be used to evaluate the composition of rare-earth-doped boro-bismuthate glass in recent amorphous structures on a broad scale. The results of this research could help researchers better understand the suitability of rare-earth doped glass samples as nuclear shielding materials.

## 2. Materials and Methods

In this study, eight different bismuth borate glass systems [30] were extensively investigated in terms of their nuclear attenuation shielding properties, along with their effective conductivity and buildup factors. The chemical composition of the studied glasses can be defined as below.

(i) 37.5Bi_2_O_3_–62.5B_2_O_3_
(ii) 38Bi_2_O_3_–60B_2_O_3_–2Al_2_O_3_
(iii) 40Bi_2_O_3_–60B_2_O_3_
(iv) 40Bi_2_O_3_–59B_2_O_3_–1Al_2_O_3_
(v) 40Bi_2_O_3_–59B_2_O_3_–1Y_2_O_3_
(vi) 40Bi_2_O_3_–59B_2_O_3_–1Nd_2_O_3_
(vii) 40Bi_2_O_3_–59B_2_O_3_–1Sm_2_O_3_
(viii) 40Bi_2_O_3_–59B_2_O_3_–1Eu_2_O_3_

In the reference study, material densities of glass samples were reported as 6.006 g/cm^3^, 6.07 g/cm^3^, 6.313 g/cm^3^, 6.233 g/cm^3^, 6.301 g/cm^3^, 6.341 g/cm^3^, 6.321 g/cm^3^, 6.328 g/cm^3^ for Sample 1, Sample 2, Sample 3, Sample 4, Sample 5, Sample 6, Sample 7 and Sample 8, respectively (see Table 1). In this section, we will present the determined nuclear radiation shielding parameters along with their technical details. Moreover, we will present a brief definition of the used Py-MLBUF online platform.

### 2.1. Nuclear Radiation Shielding Parameters

It is essential to comprehend the Lambert–Beer law [31,32], shown in Equation (1), to simplify the mass attenuation coefficient.

(1) $$I=I_{o}e^{-\mu t}$$

I: Intensity of the secondary gamma rays

I_0_: Intensity of the initial gamma rays

μ: Linear attenuation coefficient

t: Thickness of the absorber

The mass attenuation coefficient (g/cm^3^) [33,34] is a measure of how likely incident photons are to interact with matter per unit density. It is calculated using the formula demonstrated in Equation (2):

(2) µ/ρ=∑iwi(μρ)i

where μ (1/cm) is the linear attenuation coefficient, and ρ is the density of the sample, w_i_ is the weight fraction of the ith constituent of the element. The Mean Free Path (MFP cm) [35] is the average distance traveled by photons before tcollision. For any photon passing through a material, MFP is calculated using Equation (3):

(3) $$\mathrm{MFP}=\frac{1}{\mu }$$

After traveling 1 mfp through a shielding medium in an ideal narrow-beam geometry, the power of monoenergetic gamma-rays is reduced by approximately 37%. By multiplying the linear attenuation coefficient by the distance in cm between the point source and the detector, a dimensionless quantity is obtained. The term “optical thickness (OT)” refers to this dimensionless quantity. When gamma-photons traverse the shield, the OT shows how many mean free path lengths they completed. Have Value Layers (HVL cm) is described as the absorber thickness necessary to eliminate the radiation intensity to half its original value. It is calculated using Equation (4):

(4) $$\mathrm{HVL}=\frac{\ln\;\left(2\right)}{\mu }$$

Another metric that is comparable to HVL cm [36,37] is the Tenth Value Layer (TVL cm), which is defined as the thickness of absorber required to decrease the radiation intensity to one-tenth of its original value. It is computed using the following Equation (5):

(5) $$\mathrm{TVL}=\frac{\ln\;\left(10\right)}{\mu }$$

The effective conductivity C_eff_ (s/m) [38] of a shielding-material for attenuation at room temperature (300 K) is related to the effective number of electrons (N_eff_) by the following Equation (6):

(6) $$C_{\mathrm{eff}}=\left(\frac{N_{\mathrm{eff}}\;\rho \;e2\;\tau }{m_{e}}\right)\times \;10^{3}\;$$

In Equation (7), ρ, e and m_e_ denote the density of shielding materials (g·cm^−3^), the charge on an electron (C), and the electron’s rest mass (kg), respectively. τ depicts the electron’s relaxation time and is calculated using the following equation:

(7) $$\tau =\frac{h}{600\pi k}$$

where h is Planck’s constant and k is the is the Boltzmann constant. In radiation studies, the term Z_eff_ is a crucial parameter. The effective atomic number function can also be used to characterize the improvement of shielding in complex shielding materials. The effective atomic number was determined in this research using the direct method of examining the atomic and electronic cross sections.

(8) $$Z_{\mathrm{eff}}=\frac{\Sigma_{i}f_{i}A_{i}{\left(\frac{\mu }{\rho }\right)}_{i}}{\Sigma_{j}f_{j}\left(\frac{A_{j}}{Z_{j}}\right){\left(\frac{\mu }{\rho }\right)}_{j}}$$

where f_i_: fraction by mole

A_i_: atomic weight

Z_j_: Atomic number of ith constituent element

The atomic number (Z_eq_) of a shielding material is assigned exclusively by incoherent scattering. Values of Z_eq_ aid in buildup factor (BUF) calculations.

Z_eq_ values in this study were obtained using the interpolation method, demonstrated in Equation (9):

(9) $$\mathrm{Zeq}=\frac{Z_{1}\left({\mathrm{logR}}_{2}-\mathrm{logR}\right)+Z_{2}\left(\mathrm{logR}-{\mathrm{logR}}_{1}\right)}{{\mathrm{logR}}_{2}-{\mathrm{logR}}_{1}}$$

where the ratio R is the determining factor for the equivalent atomic number, for a particular photon energy.

(10) $$R=\frac{\mu_{m\;\mathrm{compton}}\;}{\mu_{m\;\mathrm{total}}}$$

Z_1_ and Z_2_ are the atomic numbers of the respective elements, which correspond to their respective ratios R_1_ and R_2_. The buildup factor is a multiplicative factor that takes into account the contributions of scattered photons to the corrected response to uncollided photons. In simpler terms, it is the ratio of incident photons to total photons. The ANS-standard was developed to compute gamma-ray BUFs for a point isotropic source operating between 0.015 and 15 MeV. The energy absorption build-up factor is a factor in which the quantity of interest is the amount of energy absorbed or stored in the interacting material. The geometric progression (G-P) fitting technique is often used to log EABFs. In this study, Py-MLBUF has been used for exposure (EBF) and energy absorption (EABF) buildup factors.

### 2.2. Python Multilayered (Py-MLBUF) Online Platform

Py-MLBUF [26] is a simple-to-use online calculator for determining gamma shielding parameters (GSP). Designed to measure the sum, 36 GSP (gamma shielding parameters) is used for gamma shielding enclosures used in radiation protection with an energy range of 0.015–15 MeV. This online platform’s feedback system was developed in the form of a question–answer format. Following sign-in, the user is prompted for the number of layers (n) in the GSE (gamma shielding energy), which is set to one for the sake of this study. The second issue concerns the composition form of the sample, which can be either elemental or compound; in this case, an elemental composition was chosen. The OT’s maximum value is set to 40 mfp by default. The energy range chosen for this analysis was 0.015–15 MeV. Last but not least, the densities and weight fractions of each substance were entered. After that, the platform calculates all of the required shielding parameters, such as MAC—mass attenuation coefficient, LAC—Linear attenuation coefficient, BUFs—buildup factor, Z_eq_—Z equivalent, Z_eff_—effective atomic number, HVL, TVL, and so on.

## 3. Results and Discussion

**Figure 1:**
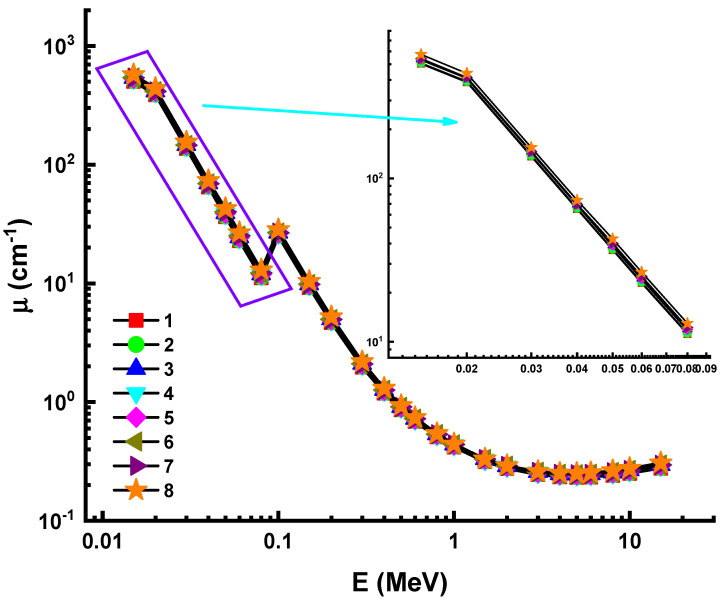
Variation of linear attenuation coefficient (µ) against Photon energy for all glasses.

The shielding properties of eight high-density glass samples were determined in terms of their gamma-ray attenuation competencies in this study. Figure 1 shows the linear attenuation coefficients (µ) of glass samples, which vary in accordance with the photon energy; the energy range is from 0.015 to 15 MeV. There are two distinct ways to get energy for photons, and both techniques need the use of electrons. Absorption may vary in two different cases: In one instance, the photon is fully absorbed; in the other, only a portion of the light is absorbed, with the remainder being scattered. There is a direct relationship between the probability of these events occurring and the temperature and photon capacity of the medium. For low-energy photons, the photoelectric effect appears to be stronger (less than 100 keV); it grows to a higher probability of occurrence as Z increases. When the energy of photons is between moderate and high, the Compton effect is significant (more than 100 keV). When an electron’s energy is more than 1.02 MeV, it will form a pair with a photon. Due to these numerous interactions, the linear attenuation coefficient may be concluded to be energy-dependent and to vary with different photon energy regions.

In Figure 1, a fast drop of 0.015 MeV to 0.08 MeV was observed. As was the case with the first field, a smooth decrement was observed in the second, demonstrating Compton scattering’s supremacy. Our results indicate that for the measured Sample 8, the highest values were discovered. This can be explained by the Eu reinforcement (Z = 63) amount in Sample 8. As is seen in Table 1, Eureinforced Sample 8 has almost the maximum glass density (i.e., 6.328 g/cm^3^). This is due to the higher atomic number of Eu compared to other additive types such as Al, Y, Nd, and Sm.

**Figure 2:**
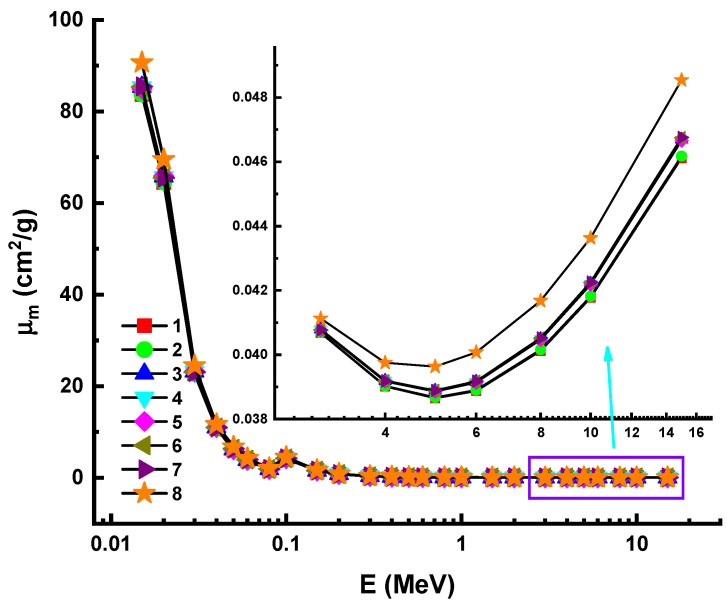
Variation of mass attenuation coefficient (µ_m_) against photon energy for all glasses. 1—37.5Bi_2_O_3_–62.5B_2_O_3_; 2—38Bi_2_O_3_–60B_2_O_3_–2Al_2_O_3_; 3—40Bi_2_O_3_–60B_2_O_3_; 4—40Bi_2_O_3_–59B_2_O_3_–1Al_2_O_3_; 5—40Bi_2_O_3_–59B_2_O_3_–1Y_2_O_3_; 6—40Bi_2_O_3_–59B_2_O_3_–1Nd_2_O_3_; 7—40Bi_2_O_3_–59B_2_O_3_–1Sm_2_O_3_; 8—40Bi_2_O_3_–59B_2_O_3_–1Eu_2_O_3_.

In the other hand, it might be more efficient to express the attenuation rate in terms of the mass of the photon-experienced target, rather than a radius. The factor affecting the attenuation rate is not the overall mass of the attenuator, but rather the region mass [39,40]. The simplest description of this phenomenon is in terms of mass attenuation coefficients (µ_m_). µ_m_ is a density-independent quantity that can be used to quantify a material’s shielding abilities. The µ_m_ values of the samples were calculated in the 0.015 MeV and 15 MeV photon energy ranges in this analysis. The distribution of m as a function of incident photon energy is depicted in Figure 2 for all glasses; µ_m_ values reported a similar attitude toward m. Additionally, the predominance of the above interactions was stated in terms of µ_m_ values. This relation is due to the seamless transitions between the densities of glass samples. Due to the fact that µ_m_ can be found by dividing by density, a similar variance pattern is predicted. However, Sample 8 had the highest µ_m_ values among the studied samples. This may be because the total mass of Sample 8 was closely related to the Eu’s Z value.

**Figure 3:**
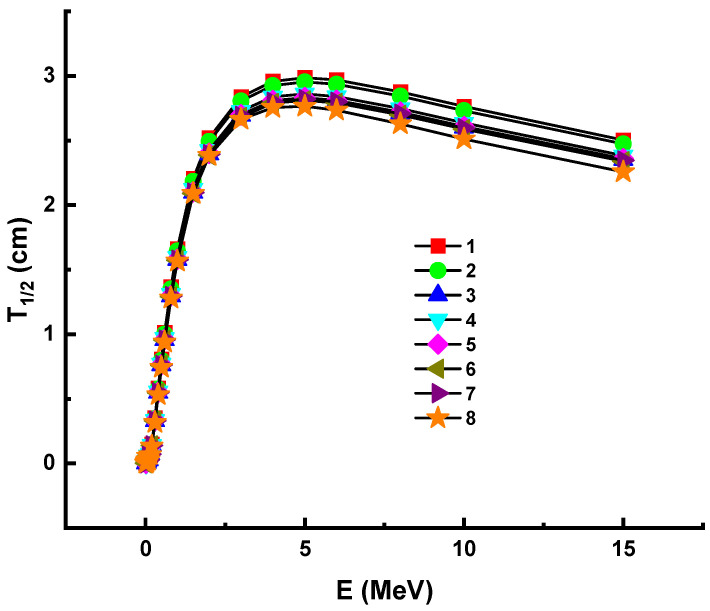
Variation of half value layer (T_1/2_) against photon energy for all glasses. 1—37.5Bi_2_O_3_–62.5B_2_O_3_; 2—38Bi_2_O_3_–60B_2_O_3_–2Al_2_O_3_; 3—40Bi_2_O_3_–60B_2_O_3_; 4—40Bi_2_O_3_–59B_2_O_3_–1Al_2_O_3_; 5—40Bi_2_O_3_–59B_2_O_3_–1Y_2_O_3_; 6—40Bi_2_O_3_–59B_2_O_3_–1Nd_2_O_3_; 7—40Bi_2_O_3_–59B_2_O_3_–1Sm_2_O_3_; 8—40Bi_2_O_3_–59B_2_O_3_–1Eu_2_O_3_.

The term HVL (T_1/2_) is important for calculating the shield thickness of a material, since it may decrease the incoming photon intensity to half its value (as 50 percent). Thus, this configuration method may be utilized to account for future practical considerations such as the optimum physical environment for shielding equipment and operational efficiency. We calculated the T_1/2_ values of investigated samples within the same energy spectrum using attenuation coefficients in this analysis. The variance of the half value layer (T_1/2_) as a feature of photon energy is shown in Figure 3.

**Figure 4:**
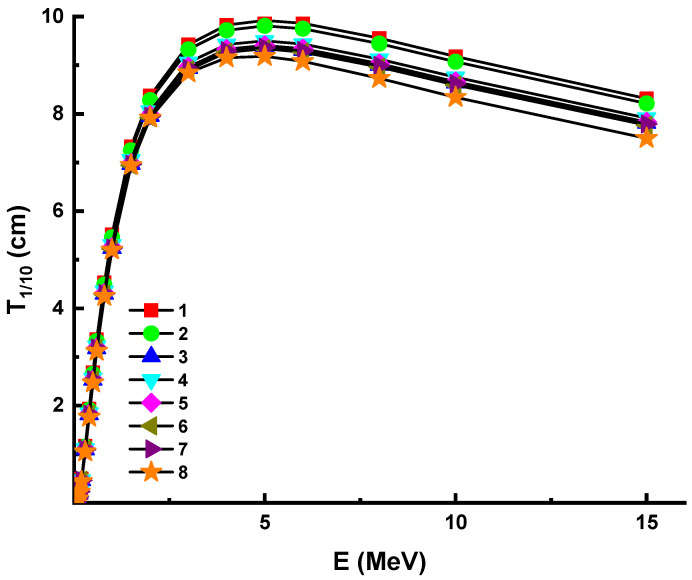
Variation of tenth value layer (T_1/10_) against photon energy for all glasses. 1—37.5Bi_2_O_3_–62.5B_2_O_3_; 2—38Bi_2_O_3_–60B_2_O_3_–2Al_2_O_3_; 3—40Bi_2_O_3_–60B_2_O_3_; 4—40Bi_2_O_3_–59B_2_O_3_–1Al_2_O_3_; 5—40Bi_2_O_3_–59B_2_O_3_–1Y_2_O_3_; 6—40Bi_2_O_3_–59B_2_O_3_–1Nd_2_O_3_; 7—40Bi_2_O_3_–59B_2_O_3_–1Sm_2_O_3_; 8—40Bi_2_O_3_–59B_2_O_3_–1Eu_2_O_3_.

With increasing photon energy, we found a direct improvement in the necessary glass thickness, which will result in a 50% reduction in incident photon density. This is demonstrated by the fact that the penetration properties of energetic photons shift as they progress from low to high energy levels [41]. In other terms, photons with a higher energy need tougher materials to achieve a halving of their power. However, it is necessary to equate the appropriate material thickness of various materials in order to halve the energy of a certain photon. As previously discussed, Sample 8 was reported with the maximum linear attenuation coefficients. Since there is an inverse relationship between T_1/2_ and linear attenuation coefficients (see Equation (5)), one can say that shields with higher linear attenuation coefficients can provide lower T_1/2_ values. This is supported by our observation that Sample 8 had the lowest T_1/2_ values of all the glasses studied. Similar to HVL, the term TVL (T_1/10_) is also a significant parameter in determining the appropriate shield thickness of a substance, since it is possible for the incoming photon intensity to be reduced to a tenth of its initial value. Consequently, this configuration function can be used for shielding products and cost effectiveness. The T_1/10_ values of investigated samples within the same energy spectrum were calculated in this analysis using attenuation coefficients. Figure 4 illustrates the variance of the tenth value layer (T_1/10_) as a characteristic of photon energy. A similar variation trend with HVL was reported for studied glass samples. The lowest T_1/10_ vales were also reported for Sample 8.

**Figure 5:**
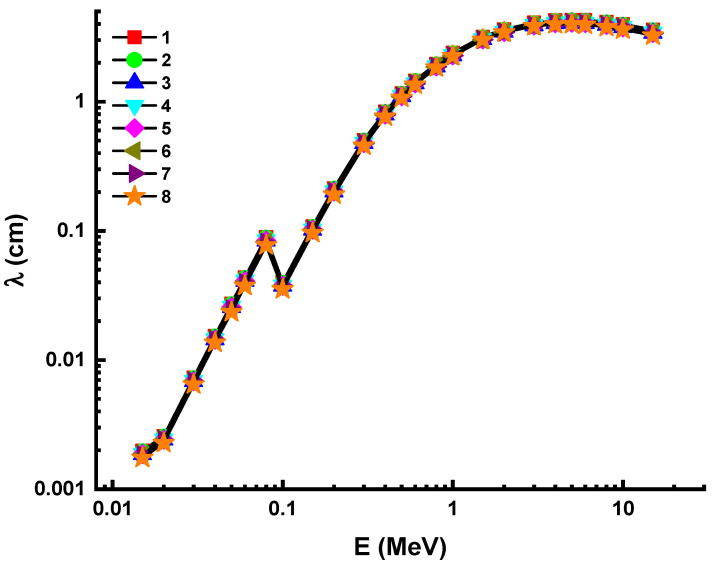
Variation of men free path (λ) against photon energy for all glasses. 1—37.5Bi_2_O_3_–62.5B_2_O_3_; 2—38Bi_2_O_3_–60B_2_O_3_–2Al_2_O_3_; 3—40Bi_2_O_3_–60B_2_O_3_; 4—40Bi_2_O_3_–59B_2_O_3_–1Al_2_O_3_; 5—40Bi_2_O_3_–59B_2_O_3_–1Y_2_O_3_; 6—40Bi_2_O_3_–59B_2_O_3_–1Nd_2_O_3_; 7—40Bi_2_O_3_–59B_2_O_3_–1Sm_2_O_3_; 8—40Bi_2_O_3_–59B_2_O_3_–1Eu_2_O_3_.

This outcome can be considered as a confirmation of the shielding effectiveness of Sample 8 in terms of halving the primary radiation intensity as well as reducing it to one tenth. MFP is the average distance traveled by photons before colliding. We determined the MFP values of studied glass samples in terms of their attenuation behaviors as a function of the average distance of an incident photon [42,43,44]. The difference in MFP as a function of photon energy is depicted in Figure 5 for all glasses.

**Figure 6:**
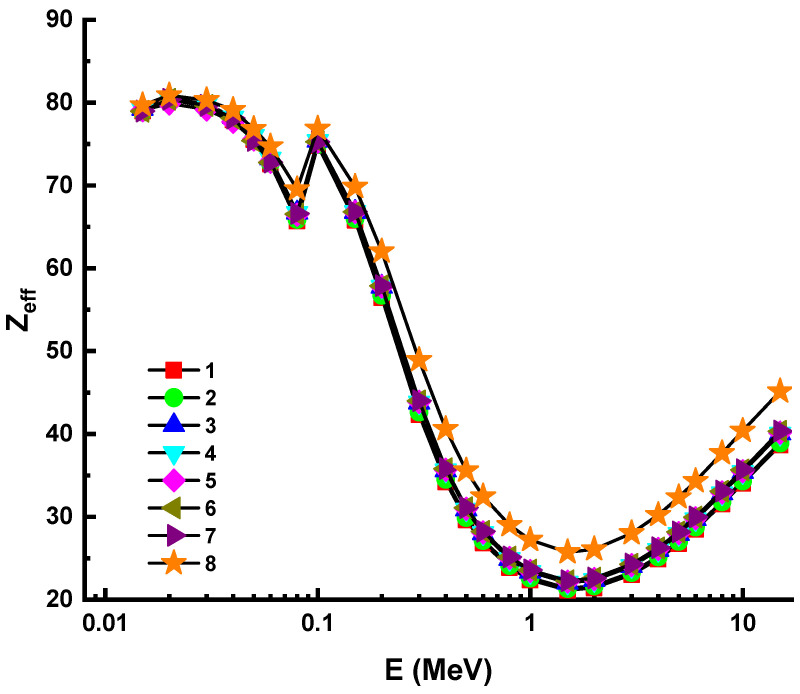
Variation of effective atomic number (Z_eff_) against Photon energy for all glasses. 1—37.5Bi_2_O_3_–62.5B_2_O_3_; 2—38Bi_2_O_3_–60B_2_O_3_–2Al_2_O_3_; 3—40Bi_2_O_3_–60B_2_O_3_; 4—40Bi_2_O_3_–59B_2_O_3_–1Al_2_O_3_; 5—40Bi_2_O_3_–59B_2_O_3_–1Y_2_O_3_; 6—40Bi_2_O_3_–59B_2_O_3_–1Nd_2_O_3_; 7—40Bi_2_O_3_–59B_2_O_3_–1Sm_2_O_3_; 8—40Bi_2_O_3_–59B_2_O_3_–1Eu_2_O_3_.

It is observed that the average travel distance of an incident gamma-ray increased as photon energy increased. This can be explained by the fact that photons have different direct penetration properties depending on their initial energy. However, Sample 8 was stated to have almost the lowest MFP values across the entire range of incident photon energies. This can be explained by the atomic structure and material density of Sample 8, as its dense atomic structure prevented these gammas from passing through. Figure 6 depicts the difference in effective atomic number values due to photon radiation. Therefore, combinations with a greater percentage of Z-valued compounds have a higher absorption rate for gamma rays. According to our results, Sample 8 has the highest Z_eff_ values across all energy levels tested. However, there was no discernible difference in Z_eff_ values. This may be clarified by the fact that the molar concentrations of glass specimens vary slightly. However, the largest difference in Z_eff_ values was observed at high energy, while the highest value was observed at 0.015 MeV in the photoelectric effect’s dominant region.

**Figure 7:**
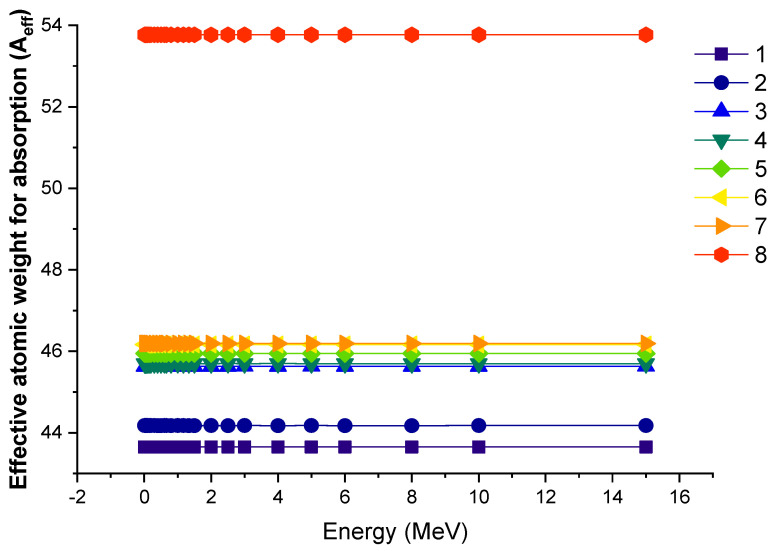
Variation of effective atomic weight (A_eff_) against photon energy for all glasses. 1—37.5Bi_2_O_3_–62.5B_2_O_3_; 2—38Bi_2_O_3_–60B_2_O_3_–2Al_2_O_3_; 3—40Bi_2_O_3_–60B_2_O_3_; 4—40Bi_2_O_3_–59B_2_O_3_–1Al_2_O_3_; 5—40Bi_2_O_3_–59B_2_O_3_–1Y_2_O_3_; 6—40Bi_2_O_3_–59B_2_O_3_–1Nd_2_O_3_; 7—40Bi_2_O_3_–59B_2_O_3_–1Sm_2_O_3_; 8—40Bi_2_O_3_–59B_2_O_3_–1Eu_2_O_3_.

Figure 7 illustrates the difference in effective atomic weight for absorption (A_eff_) as a function of photon energy for all glasses. The changes in the overall atomic weight of the mixture are directly related to the additive properties, which resulted in densities ranging from 6.006 g/cm^3^ to 6.328 g/cm^3^. As illustrated in Figure 7, the additive types in the glass samples had an effect on the effective atomic weight for photon absorption. As a result, Sample 8 was reported to have the highest A_eff_ value of the examined glasses.

**Figure 8:**
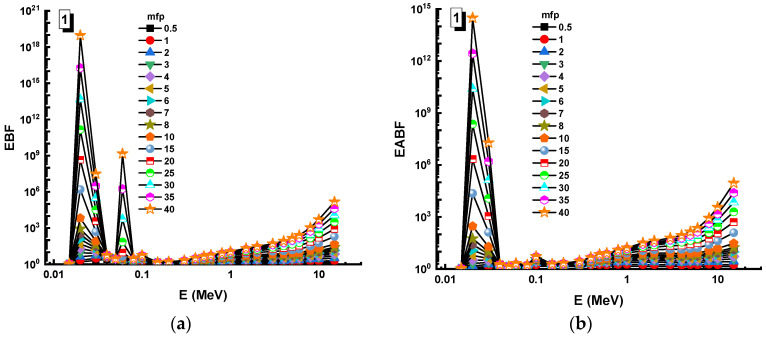
Variation of (**a**) EBF and (**b**) EABF against photon energy for Sample 1.

**Figure 9:**
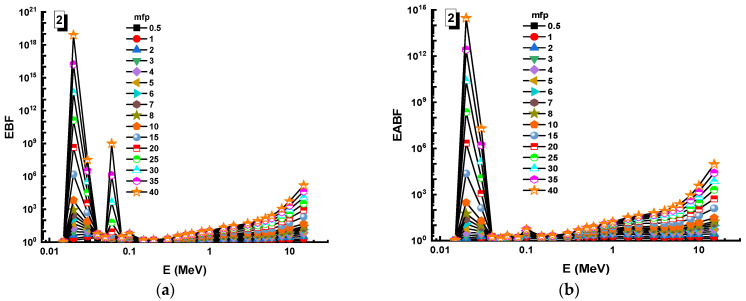
Variation of (**a**) EBF and (**b**) EABF against photon energy for Sample 2.

**Figure 10:**
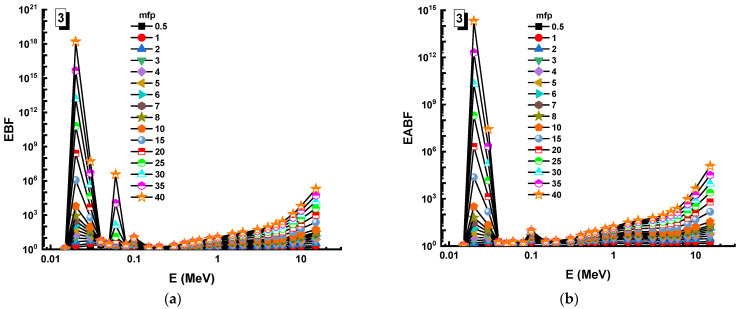
Variation of (**a**) EBF and (**b**) EABF against photon energy for Sample 3.

**Figure 11:**
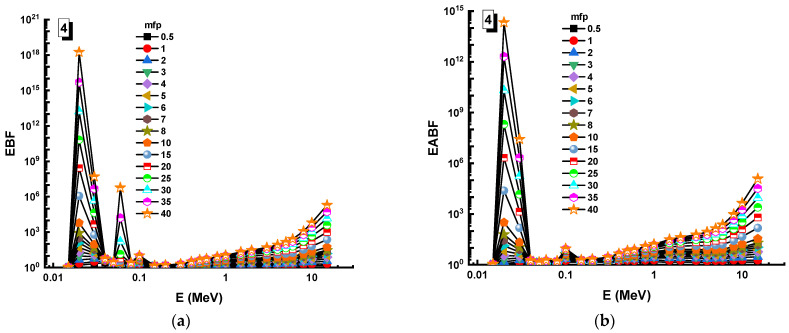
Variation of (**a**) EBF and (**b**) EABF against photon energy for Sample 4.

**Figure 12:**
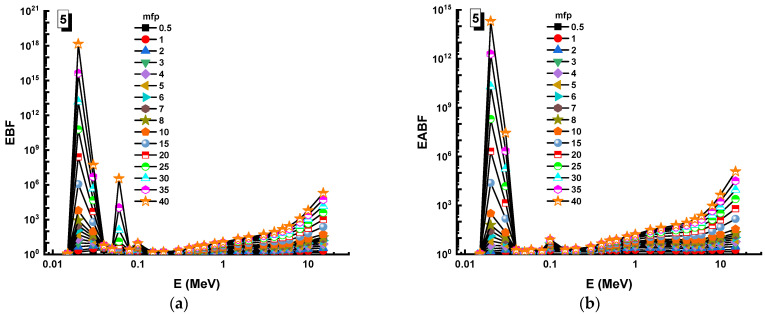
Variation of (**a**) EBF and (**b**) EABF against photon energy for Sample 5.

**Figure 13:**
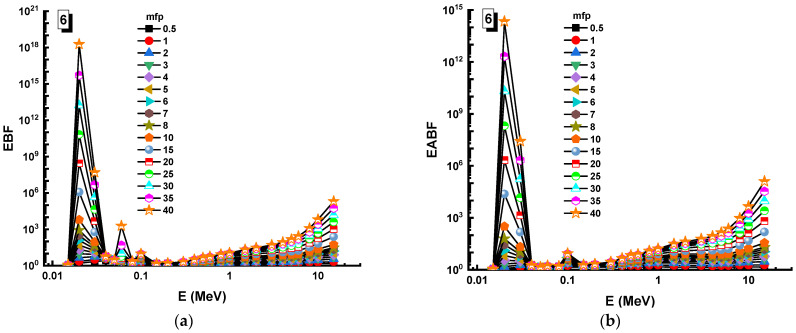
Variation of (**a**) EBF and (**b**) EABF against photon energy for Sample 6.

**Figure 14:**
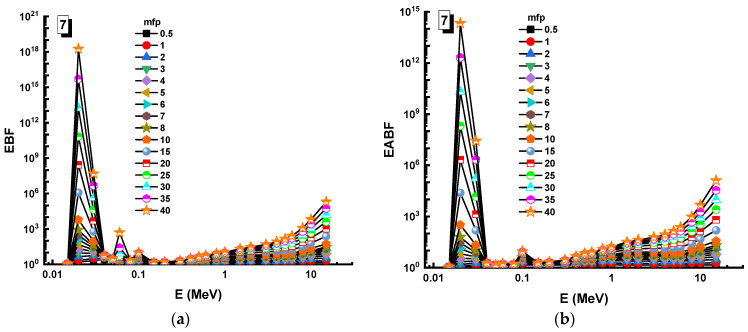
Variation of (**a**) EBF and (**b**) EABF against photon energy for Sample 7.

**Figure 15:**
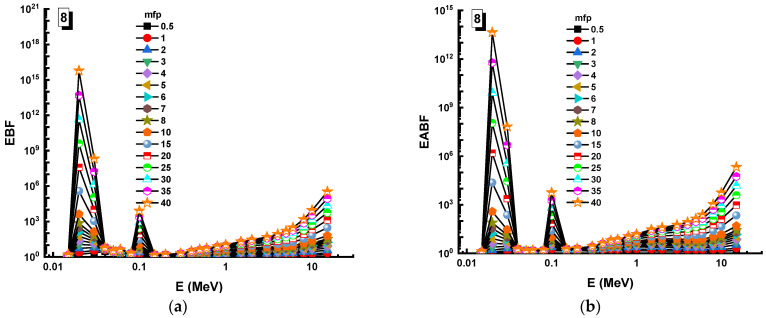
Variation of (**a**) EBF and (**b**) EABF against photon energy for Sample 8.

In this analysis, the EBF and EABF values were determined using the geometry progressive (G-P) method. As a result, the difference between the exposure buildup factor (EBF) and the energy absorption buildup factor (EABF) for glass samples with penetration depths of 0.5, 1, 2, 3, 4, 5, 6, 7, 8, 9, 10, 15, 20, 25, 30, and 40 mfp has been visualized in Figure 8, Figure 9, Figure 10, Figure 11, Figure 12, Figure 13, Figure 14 and Figure 15. Other than the variance in EBF values, Sample 8 was determined to have the highest EBF values. In the interaction of the product and the detector material, the amount of energy in the product affects the quantity that is influenced by the amount of energy in the product and the detector function. Concerning the whole region of concentration, it is called exposure, and in the air, a detector reacts to that which is being absorbed. In EABF, the results indicated a similar attitude toward EBF; the Sample 8 EABF values were also captured as a consequence. In the 0.015–15 MeV photon energy range, the effective conductivity Ceff (s/m) values of the investigated glasses were finally obtained. The effective conductivity C_eff_ (s/m) of a shielding material for attenuation at room temperature (300 K) is related to the effective number of electrons. This parameter might provide useful information about investigated materials in terms of their attenuation condition at room temperature.

**Figure 16:**
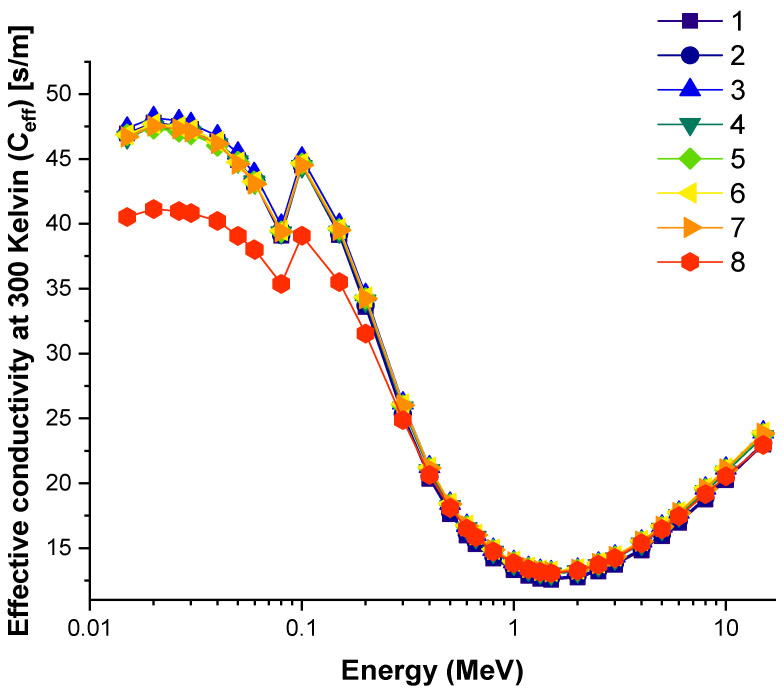
Variation of effective conductivity (C_eff_) at 300K (s/m) for all glasses.

Figure 16 shows variation of effective conductivity (C_eff_) at 300 K (s/m) for all glasses. The results imply that as incoming photon energy rises, the effective thermal conductivity quickly falls, particularly in the low energy region, where the photoelectric effect dominates the photon–matter interaction [45,46]. However, Sample 8 was found to have the lowest C_eff_ values of all the samples examined at low energies. The findings clearly reflect that Sample 8 has the minimum heat conductivity at all photon energies, which can also be considered another beneficial feature of Sample 8 in terms of radio-protective properties

## 4. Conclusions

Currently, a fascinating field of study is the fabrication of optical instruments using rare-earth element-doped materials. Rare-earth element-doped glasses are frequently used for a variety of uses, including laser materials, optical amplifiers, optical memory systems, magneto-optical instruments, surgical lasers, eye-safe lasers, flat panel displays, fluorescent lighting, and white led lasers. In addition to all these advantages, glasses doped with rare-earth oxides are used effectively in radiation shielding studies due to their high densities and their ability to decrease the number of non-bridging oxygens in the glass network that they are doped to, and thus result in a more compact structure and increased density in the newly formed material. In our study, eight different bismuth borate glasses were investigated in terms of their nuclear attenuation shielding properties, along with effective conductivity and buildup factors, for potential use in nuclear radiation facilities. Our research uncovered the following key points;

(i) The µ_m_ values of 64.49 cm^2^/g, 64.59 cm^2^/g, 65.79 cm^2^/g, 65.70 cm^2^/g, 65.87 cm^2^/g, 65.49 cm^2^/g, 65.52 cm^2^/g, 69.48 cm^2^/g were recorded for Sample 1, Sample 2, Sample 3, Sample 4, Sample 5, Sample 6, Sample 7 and Sample 8 glasses at 0.015 MeV, respectively. Generally, the µ_m_ values obeyed the trend: (µ_m_)_8_ > (µ_m_)_7_ > (µ_m_)_6_ > (µ_m_) _5_ > (µ_m_) _4_ > (µ_m_)_3_ > (µ_m_) _2_ > (µ_m_) _1_
(ii) The T_1/2_ values of 2.5193 cm, 2.4923 cm, 2.3943 cm, 2.4251 cm, 2.4004 cm, 2.3857 cm, 2.3934 cm, 2.3831 cm were recorded Sample 1, Sample 2, Sample 3, Sample 4, Sample 5, Sample 6, Sample 7 and Sample 8 glasses at 2 MeV, respectively.
(iii) The findings indicated that Sample 8 had the lowest values of MFP at the photon energies investigated.
(iv) The findings indicate that Sample 8 has the highest Z_eff_ values at all energy levels examined.
(v) Sample 8 was reported as having the maximum EBF and EABF values, while the minimum values were reported for the Sample 1.

From the results, it can be concluded that the effect of Eu is higher than other types of rare-earth elements such as Y, Nd, Sm and Al in terms of radiation attenuation competencies and effective conductivity at 300 K. The literature review showed that researchers are considering different types of investigations in terms of adding rare-earth for nuclear radiation shielding improvements. As a result of the findings, the scientific community will be able to determine the most appropriate additive rare-earth types and glass compositions to offer the aforementioned shielding characteristics in terms of demands and usage criteria. Additionally, advanced research such as mechanical and thermal analyses of these glasses are suggested in order to get a better knowledge of their overall appropriateness for shielding applications in various kinds of radiation facilities.

## Figures and Tables

**Table 1:** The codes, elemental compositions, weight fractions and densities of the studied glass samples.

Sample Code	B	O	Bi	Al	Y	Nd	Sm	Eu	Density (g/cm^3^)
1	0.061915	0.219923	0.718162	-	-	-	-	-	6.006
2	0.058731	0.217306	0.719077	0.004886	-	-	-	-	6.07
3	0.056857	0.210372	0.732771	-	-	-	-	-	6.313
4	0.05583	0.210074	0.731734	0.002362	-	-	-	-	6.233
5	0.055529	0.208942	0.727789	-	0.007741	-	-	-	6.301
6	0.055263	0.20794	0.7243	-	-	0.012498	-	-	6.341
7	0.055233	0.20783	0.723916	-	-	-	0.013021	-	6.321
8	0.05523	0.20780	0.72382	-	-	-	-	0.00658	6.328

## Data Availability

Data is contained within the article.
